# Timing Matters: A Randomized Controlled Trial Comparing Preoperative and Postoperative Erector Spinae Plane Block for Analgesia in Laparoscopic Cholecystectomy

**DOI:** 10.3390/medicina61101806

**Published:** 2025-10-09

**Authors:** Mehmet Sait Acar, Veli Fahri Pehlivan, Basak Pehlivan, Erdogan Duran

**Affiliations:** 1 Department of Anesthesia and Reanimation, Ministry of Health, Akçakale State Hospital, 63200 Sanliurfa, Türkiye; canacar44@gmail.com; 2 Department of Anesthesia and Reanimation, Faculty of Medicine, Osmanbey Campus, Harran University, 63300 Sanliurfa, Türkiye; bpehlivan@harran.edu.tr (B.P.); dreduran@harran.edu.tr (E.D.)

**Keywords:** erector spinae plane block (ESPB), laparoscopic cholecystectomy (LC), timing, postoperative pain, hemodynamic stability, regional anesthesia, ERAS (enhanced recovery after surgery)

## Abstract

*Background and Objectives:* The erector spinae plane block (ESPB) is an emerging regional anesthesia technique that has demonstrated effectiveness in reducing postoperative pain and opioid consumption following laparoscopic cholecystectomy (LC). However, the optimal timing of ESPB whether administered preoperatively or postoperatively remains uncertain, particularly regarding its influence on intraoperative hemodynamic stability and procedural feasibility. This study aimed to compare the analgesic efficacy, intraoperative hemodynamic profiles, and procedural advantages of preoperative versus postoperative ESPB in patients undergoing elective LC. *Materials and Methods:* In this prospective, randomized, and single-blind clinical trial, 80 ASA I–II adult patients scheduled for elective LC were randomly assigned to receive bilateral ESPB either before anesthesia induction (Group 1) or immediately after surgery but prior to extubation (Group 2). All patients received standardized general anesthesia. The primary outcome was postoperative pain measured by the numeric rating scale (NRS) at 2 h postoperatively. Secondary outcomes included NRS scores at other time points (0, 4, 6, 12, and 24 h), intraoperative and postoperative hemodynamic parameters, cumulative 24 h rescue analgesic consumption, patient satisfaction scores, and adverse events. *Results:* Both groups experienced significant reductions in postoperative NRS scores, with no statistically significant differences between groups in pain intensity or tramadol consumption. However, the preoperative ESPB group exhibited significantly more stable intraoperative blood pressure readings, particularly at 30 and 60 min after incision and at extubation. No ESPB-related complications occurred in either group. Patient satisfaction levels were comparable across groups. *Conclusions:* Preoperative and postoperative ESPBs offer comparable analgesic efficacy and opioid sparing effects in LC. However, preoperative ESPB provides enhanced intraoperative hemodynamic stability and avoids the logistical challenges of performing blocks under anesthesia, including repositioning related risks. These findings suggest that preoperative ESPB may be considered for integration into enhanced recovery after surgery (ERAS) protocols for minimally invasive biliary surgery, pending further large-scale multicenter trials.

## 1. Introduction

Laparoscopic cholecystectomy (LC) is a common minimally invasive procedure for gallstone disease, yet postoperative pain remains a challenge, arising from incisional, visceral, and referred sources [1]. Effective pain management is crucial to enhance recovery, reduce opioid use, and improve patient outcomes [2,3]. Regional anesthesia, as part of multimodal analgesia, has gained traction for its opioid-sparing effects [4].

The erector spinae plane block (ESPB), described in 2016, involves injecting local anesthetic between the erector spinae muscle and transverse process, targeting spinal nerve rami to provide somatic and visceral analgesia [5]. Its ultrasound-guided approach minimizes complications, making it suitable for abdominal surgeries like LC [6,7]. Studies have demonstrated ESPB’s efficacy in reducing pain and analgesic requirements in LC [8,9]. However, the optimal timing of ESPB administration (whether before or after surgery) remains an open question, as a focused review of the literature revealed a paucity of high-quality comparative data evaluating the timing dependent efficacy and safety of this technique.

From a procedural standpoint, preoperative ESPB offers several clinical advantages. Administering the block before the induction of anesthesia facilitates performance in a fully conscious or lightly sedated patient, which allows optimal positioning (e.g., lateral decubitus or prone) without compromising hemodynamics or airway safety [10,11]. This approach also enables preemptive analgesia, which may attenuate the intraoperative stress response and promote hemodynamic stability. Conversely, performing ESPB postoperatively necessitates repositioning the patient while under residual anesthesia or in early recovery. This may be technically more challenging and carries potential risks, particularly in patients with difficult airways, obesity, or impaired respiratory reserve [12,13]. Furthermore, delayed block administration may reduce the effectiveness of preemptive analgesia and limit its hemodynamic benefits. However, despite these theoretical advantages and clinical considerations, the current literature comparing preoperative versus postoperative ESPB in terms of safety, feasibility, and analgesic efficacy remains limited, warranting further investigation.

Therefore, the primary aim of this prospective randomized study was to compare the analgesic efficacy of preoperative versus postoperative erector spinae plane block (ESPB) in patients undergoing laparoscopic cholecystectomy (LC). Our primary hypothesis was that preoperative ESPB would result in superior postoperative pain control compared with postoperative ESPB due to its preemptive analgesic effect. The primary outcome was postoperative pain intensity measured by the numeric rating scale (NRS) at 2 h postoperatively. Secondary outcomes included intraoperative and postoperative hemodynamic parameters (heart rate and blood pressure), cumulative 24 h tramadol consumption, patient-reported satisfaction scores, and adverse events. By addressing both analgesic effectiveness and perioperative physiological responses, this study aims to inform optimal ESPB timing in clinical anesthesia practice.

## 2. Materials and Methods

### 2.1. Study Design

**Figure 1:**
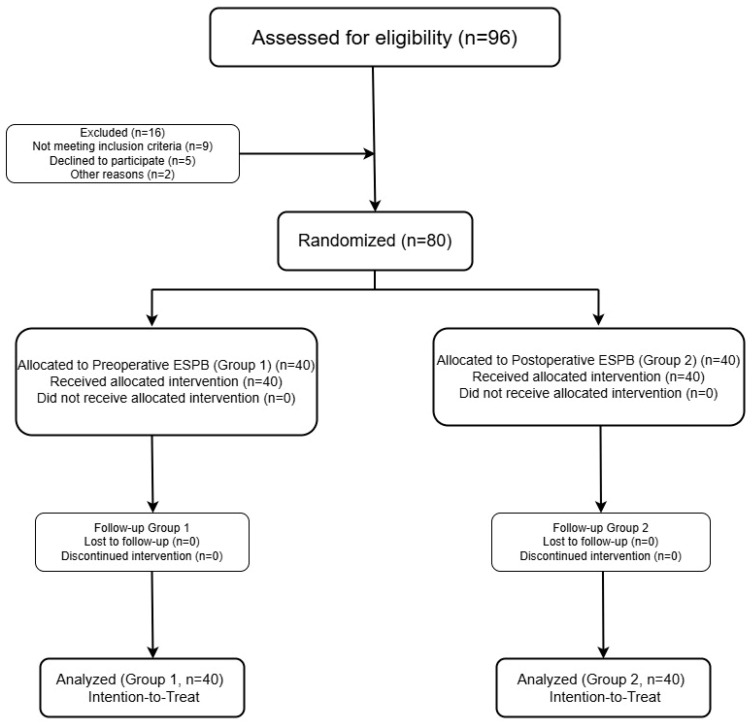
CONSORT flow diagram of patient enrollment and randomization.

This prospective, randomized, single-blind, and parallel group clinical trial was conducted at the Department of Anesthesiology and Reanimation, Harran University Faculty of Medicine, between 15 January and 11 November 2024. Ethical approval was obtained from the Harran University Clinical Research Ethics Committee (decision no: HRÜ/2023.17.13, dated 18 September 2023), and this trial was registered in ClinicalTrials.gov (identifier: NCT06670313). All procedures were conducted in accordance with the Declaration of Helsinki. Written informed consent was obtained from all participants prior to enrollment. This study adhered to the CONSORT (Consolidated Standards of Reporting Trials) guidelines throughout its design, implementation, and reporting (Figure 1).

### 2.2. Sample Size and Power Calculation

The sample size was determined based on the findings of a previous randomized controlled trial by Altiparmak et al. [9], which demonstrated a significant reduction in postoperative pain scores following ESPB. Using an effect size of Cohen’s d = 0.71, a priori power analysis was performed via G*Power 3.1. With a two-tailed alpha of 0.05 and 80% statistical power, the minimum required sample size was calculated as 32 patients per group. To account for potential dropouts and ensure statistical robustness, 80 patients were enrolled (40 per group).

### 2.3. Participants

Of the 96 patients initially assessed for eligibility, 16 were excluded: 9 did not meet the inclusion criteria, 5 declined to participate, and 2 were excluded for other reasons. (Figure 1) The final study cohort consisted of 80 adult patients (aged 18–65 years) classified as ASA physical status I–II and scheduled for elective laparoscopic cholecystectomy (LC). Inclusion criteria were adult patients (18–65 years) with ASA physical status I-II scheduled for elective LC. Exclusion criteria included refusal to participate, allergy to local anesthetics, coagulopathy, infection at the injection site, or serious systemic diseases that could interfere with study compliance or outcomes.

### 2.4. Randomization and Blinding

Eligible patients were randomized at a 1:1 ratio into two parallel groups using a computer-generated random sequence.

- Group 1 (*n* = 40): received bilateral ESPB in the preoperative period prior to induction of anesthesia,
- Group 2 (*n* = 40): received bilateral ESPB postoperatively prior to extubation.

All patients received the intervention as allocated. No participants were lost to follow-up or discontinued the intervention. While the anesthesiologist administering the block was unblinded due to the nature of the procedure, patients remained blinded to group assignment and timing of the ESPB intervention. Patient blinding was maintained by informing patients that they would receive the block at some point during their perioperative care, without specifying the exact timing. This approach minimized the risk of unblinding while acknowledging the practical limitations of blinding the intervention itself. Although double blinding was not feasible due to the nature of the ESPB technique, patient blinding was rigorously maintained to minimize assessment bias in subjective outcomes such as pain and satisfaction. The assessors for NRS and satisfaction scores were also blinded to group assignment.

### 2.5. Anesthetic Management and ESPB Protocol

Upon arrival in the operating room, standard monitoring including electrocardiography, pulse oximetry, non-invasive blood pressure, and end-tidal CO_2_ (EtCO_2_) was initiated for all patients. Sedation was provided preoperatively with 2 mg of midazolam intravenously. General anesthesia was induced with fentanyl (1.5 µg/kg), propofol (2–3 mg/kg), and rocuronium (0.6 mg/kg). After tracheal intubation, patients were ventilated with a fresh gas flow of 2 L/min using a 50:50 mixture of oxygen and air, targeting an EtCO_2_ range of 34–39 mmHg. Anesthesia was maintained with sevoflurane at 1 MAC and a remifentanil infusion.

At the end of surgery, 2 mg/kg of sugammadex was administered intravenously to reverse neuromuscular blockade. Patients with adequate spontaneous respiration and verbal responsiveness were extubated and transferred to the post-anesthesia care unit (PACU). Discharge from the PACU to the ward was based on an Aldrete score ≥ 9. All patients received intravenous paracetamol (10 mg/kg) every 8 h postoperatively as part of the standard analgesic regimen. Hemodynamic parameters (systolic, diastolic, and mean arterial pressure), heart rate, and NRS pain scores were recorded at postoperative 0, 2, 4, 6, 12, and 24 h. Intraoperative hemodynamic measurements were recorded at 0, 15, 30, and 60 min.

### 2.6. Preoperative ESPB Protocol (Group 1)

After initial monitoring, patients in Group 1 underwent bilateral erector spinae plane block (ESPB) at the T6–T8 vertebral level before general anesthesia induction. Following aseptic preparation with 10% povidone-iodine, ESPB was performed under ultrasound guidance by a single experienced anesthesiologist (Dr. O.B.) not involved in this study. A linear high-frequency ultrasound probe was used to identify the transverse processes, then shifted 3 cm laterally to locate the fascial plane deep to the erector spinae muscle. Using a 21G 100 mm block needle (Pajunk^®^, Geisingen, Germany), saline hydrodissection was first applied to confirm correct needle placement. Subsequently, 10 mL of 0.5% bupivacaine, 5 mL of 2% lidocaine, and 5 mL of 0.9% saline were injected on each side, yielding a total volume of 40 mL bilaterally. General anesthesia was induced immediately following block administration.

### 2.7. Postoperative ESPB Protocol (Group 2)

In Group 2, general anesthesia was first induced using the same protocol described above. At the conclusion of the laparoscopic cholecystectomy, and prior to extubation, patients were repositioned into the lateral decubitus position. Following antiseptic preparation with 10% povidone-iodine, bilateral ESPB was performed at the T6–T8 levels by the same experienced anesthesiologist (Dr. O.B.) using identical ultrasound and needle techniques as in Group 1. A total of 40 mL (20 mL per side) of local anesthetic solution was administered, consisting of 10 mL of 0.5% bupivacaine, 5 mL of 2% lidocaine, and 5 mL of saline per side. Patients were subsequently returned to the supine position and extubated per protocol.

**Figure 2:**
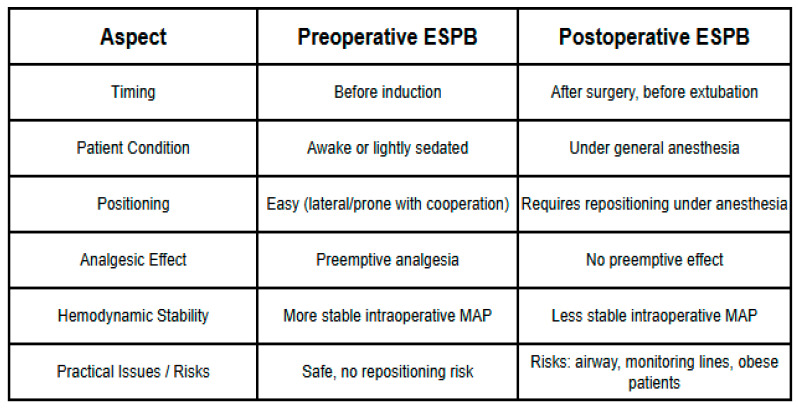
Workflow comparison of preoperative versus postoperative erector spinae plane block (ESPB) in laparoscopic cholecystectomy.

This schematic table summarizes the key procedural differences between preoperative and postoperative ESPB (Figure 2). Preoperative ESPB is performed before induction in awake or lightly sedated patients, enabling optimal positioning, preemptive analgesia, and greater intraoperative hemodynamic stability without the risks of repositioning. In contrast, postoperative ESPB is performed under general anesthesia at the end of surgery, requiring repositioning that may compromise airway safety and monitoring lines, particularly in high-risk or patients with obesity. The figure highlights these practical and physiological considerations, which are critical for interpreting outcomes and planning ERAS-based perioperative strategies.

### 2.8. Outcome Measures

The primary outcome was the assessment of postoperative pain using the numeric rating scale (NRS, 0: no pain to 10: worst pain imaginable) at 2 h postoperatively.

Secondary outcomes included the following:

- NRS scores at other time points: postoperative 0 (PACU), 4, 6, 12, and 24 h.
- Hemodynamic parameters (heart rate, systolic and diastolic blood pressure, and mean arterial pressure) at specified intraoperative and postoperative time points.
- Cumulative rescue analgesic use within 24 h (tramadol 1 mg/kg IV, administered as needed).
- Patient satisfaction at 24 h (measured on a 5-point Likert scale).
- Adverse events such as pneumothorax, local anesthetic systemic toxicity (LAST), nausea, and vomiting.

### 2.9. Statistical Analysis

All statistical analyses were performed using SPSS version 25.0 (IBM Corp., Armonk, NY, USA). Descriptive statistics for continuous variables were expressed as median (interquartile range [IQR]) for skewed distributions and mean ± standard deviation (SD) for normally distributed data, and categorical variables as frequency and percentage. Normality of distribution was assessed using the Shapiro–Wilk test. Between-group comparisons were performed using the independent samples *t*-test or Mann–Whitney U test for continuous variables and the chi-square or Fisher’s exact test for categorical variables. For ordinal outcomes such as NRS scores and Likert satisfaction, appropriate non-parametric tests (e.g., Mann–Whitney U test) were used. For outcomes where baseline characteristics showed significant imbalance or trends (e.g., comorbidity prevalence, BMI), covariate-adjusted analyses (e.g., ANCOVA or mixed models) were considered to account for potential confounding effects. A *p*-value of <0.05 was considered statistically significant.

## 3. Results

### 3.1. Baseline Demographic and Clinical Characteristics of the Study Population

The demographic and clinical characteristics of the 80 patients enrolled in this study are summarized in Table 1. No statistically significant differences were observed between Group 1 and Group 2 in terms of age (45.4 ± 14.2 vs. 48.0 ± 13.6 years; *p* = 0.413) or body mass index (BMI) (26.1 ± 3.92 vs. 27.6 ± 3.00 kg/m^2^; *p* = 0.054). The proportion of female patients was comparable in both groups (72.5% vs. 70.0%; *p* = 0.805). While the presence of comorbidities did not significantly differ statistically between groups (57.5% vs. 77.5%; *p* = 0.171), a trend towards higher comorbidity in Group 2 was noted. Similarly, BMI showed a trend towards higher values in Group 2 (*p* = 0.054). Standardized mean differences (SMDs) for these variables were calculated to assess the magnitude of imbalance, and where SMDs indicated a meaningful difference (>0.2), these variables were considered as covariates in adjusted analyses. The most common comorbid conditions were hypertension and diabetes mellitus, either alone or in combination. Among the baseline characteristics collected were chronic medication use, Charlson comorbidity index, ASA score, type of surgical procedure (all elective LC), underlying disease (cholelithiasis/cholecystitis), laboratory parameters (hemoglobin, white blood cell count), smoking habits, and duration of surgery. These findings indicated that baseline characteristics were generally well balanced between the study groups, and relevant trends were accounted for in the statistical adjustments. As the differences did not reach statistical significance, these data were not presented in tabular form.

### 3.2. Effect of ESPB Timing on Postoperative Pain Intensity: NRS Score Trajectory

Postoperative NRS scores (mean ± SD) at various time points are presented in Table 2. Both groups demonstrated significant reductions in NRS scores over time. No statistically significant differences were observed between Group 1 and Group 2 in NRS scores at the primary endpoint (2 h postoperatively) or at any other time point (*p* > 0.05 for all comparisons).

### 3.3. Effect of ESPB Timing on Intraoperative and Postoperative Hemodynamic Profiles

Intraoperative hemodynamic parameters (mean ± SD) are summarized in Table 3. The preoperative ESPB group (Group 1) exhibited significantly more stable intraoperative blood pressure readings compared with Group 2. Specifically, mean arterial pressure (MAP) was significantly higher in Group 1 at 30 min (*p* < 0.01) and 60 min (*p* < 0.05) after incision, and at extubation (*p* < 0.01). Heart rate differences were not statistically significant between groups at any intraoperative time point.

Postoperative hemodynamic parameters (mean ± SD) are summarized in Table 4. No significant differences were observed between groups in heart rate, systolic blood pressure, diastolic blood pressure, or mean arterial pressure at any postoperative time point (*p* > 0.05 for all comparisons).

**Figure 3:**
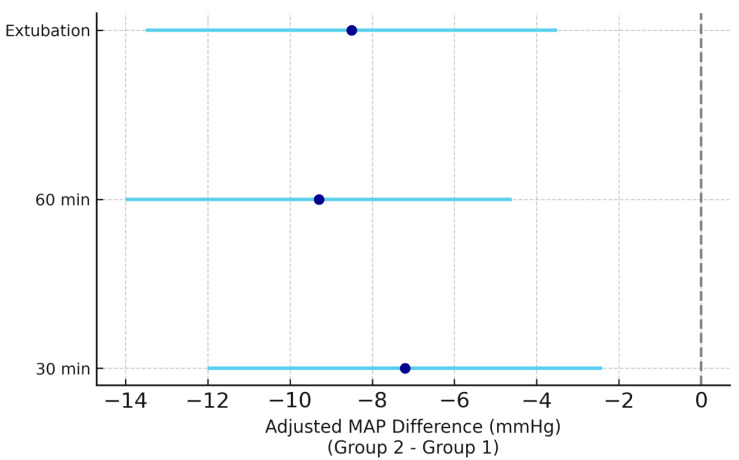
Forest plot of adjusted mean arterial pressure (MAP) differences between preoperative and postoperative ESPB groups.

Figure 3 illustrates adjusted MAP differences over time between groups using a forest plot. 

The figure shows adjusted intraoperative MAP differences (Group 2–Group 1) at 30 min, 60 min, and extubation. Points represent mean differences and horizontal bars indicate 95% confidence intervals. Negative values favor preoperative ESPB, suggesting more stable hemodynamics during surgery compared with postoperative administration.

### 3.4. Effect of ESPB Timing on the Incidence and Dose of Rescue Tramadol Use

The need for supplemental analgesics was evaluated at 0, 2, 4, 6, 12, and 24 h postoperatively in patients reporting an NRS score of 5 or higher. As shown in Table 5, there were no statistically significant differences between the preoperative (Group 1) and postoperative (Group 2) ESPB groups at any time point in terms of additional analgesic requirement (*p* > 0.05 for all comparisons). The mean cumulative dose of rescue tramadol was slightly higher in Group 1 (30.0 ± 45.9 mg) than in Group 2 (22.5 ± 41.8 mg); however, this difference did not reach statistical significance (*p* = 0.062).

### 3.5. Impact of ESPB Administration Timing on 24-Hour Patient Satisfaction

At 24 h postoperatively, a 5-point Likert scale was used to assess patient satisfaction regarding postoperative analgesia. As shown in Table 6, no statistically significant difference in satisfaction scores was observed between Group 1 and Group 2 (*p* = 0.191). The majority of patients in both groups reported high levels of satisfaction, with 75% of Group 1 and 65% of Group 2 rating their experience as either “very satisfactory” or “satisfactory”. Only a small proportion of patients in Group 2 (10%) reported dissatisfaction (Table 6).

### 3.6. Adverse Events

No ESPB-related complications such as pneumothorax or local anesthetic systemic toxicity (LAST) were observed in either group. The incidence of postoperative nausea and vomiting (PONV) was also comparable between Group 1 (10%) and Group 2 (12.5%) (*p* = 0.75).

## 4. Discussion

This randomized controlled trial investigated the optimal timing of erector spinae plane block (ESPB) for analgesia in laparoscopic cholecystectomy (LC), comparing preoperative versus postoperative administration. Our findings indicate that both approaches provide comparable postoperative pain control and opioid-sparing effects. However, preoperative ESPB demonstrated a significant advantage in maintaining intraoperative hemodynamic stability. This study addresses a critical gap in the literature by providing high-quality comparative data on the timing-dependent efficacy and safety of ESPB in LC, particularly concerning its impact on perioperative physiological responses.

The comparable analgesic efficacy between preoperative and postoperative ESPB observed in our study is consistent with some recent reports but differs from others. For example, a meta-analysis by Chauhan et al. [14] evaluating ESPB timing in abdominal surgeries demonstrated no significant differences in overall pain scores, which supports our findings. In contrast, several studies have suggested that preoperative ESPB may provide slightly superior early postoperative pain control [15,16]. Such discrepancies may reflect variations in surgical techniques, anesthetic regimens, or patient characteristics. We selected the 2 h postoperative time point as the primary endpoint, as this period typically corresponds to peak pain intensity after LC and aligns with the expected duration of the local anesthetic administered.

A key finding of our study is the enhanced intraoperative hemodynamic stability observed in the preoperative ESPB group, likely reflecting attenuation of the surgical stress response through preemptive analgesia. Patients receiving ESPB before incision demonstrated significantly more stable systolic and mean arterial pressures at critical intraoperative time points (30 and 60 min) and at extubation, while postoperative parameters showed no significant group differences. These results are clinically relevant, as hemodynamic fluctuations during surgery may predispose patients with cardiovascular comorbidities to adverse outcomes. Our findings align with prior evidence indicating that regional blocks can mitigate intraoperative stress responses and reduce the need for vasoactive agents, thereby lowering perioperative risk [11,17]. For example, Liao et al. reported that ESPB enhanced hemodynamic stability and decreased analgesic requirements in the surgical stabilization of rib fractures [11], while Chen et al. demonstrated improved perioperative hemodynamics with ESPB during transforaminal lumbar interbody fusion [17]. Collectively, this evidence underscores the physiological and clinical advantages of administering ESPB preoperatively.

From a procedural standpoint, performing ESPB preoperatively in conscious or lightly sedated patients facilitates optimal positioning and eliminates the risks associated with postoperative repositioning under residual anesthesia. Repositioning in the recovery phase may cause physiological disturbances, such as hypotension or hypertension, and risks displacement of monitoring lines or airway devices [10,13,18,19,20]. Additionally, it can be hazardous in patients with obesity or those with compromised respiratory reserves [18,21,22]. The ability to perform ESPB preoperatively with patient cooperation not only enhances safety and comfort but also allows real-time feedback during block placement [18,19]. In our study, block consistency was further ensured as all procedures were performed by a single experienced anesthesiologist. Although we did not specifically quantify the incidence of airway- or position-related complications, the theoretical and practical advantages of preoperative ESPB in mitigating these risks are substantial and support its consideration as the preferred timing in clinical practice.

The concept of preemptive analgesia, where an analgesic intervention is initiated before the noxious stimulus, is a key theoretical advantage of preoperative ESPB. Although our study did not find statistically significant differences in postoperative pain scores or rescue analgesic consumption between the two groups, the trend towards lower NRS scores in the preoperative group at some time points, coupled with the observed hemodynamic stability, suggests a potential preemptive effect. This aligns with the principle that blocking nociceptive pathways before surgical incision can reduce central sensitization and improve postoperative pain control [2,3]. The preemptive effect of ESPB has been demonstrated in other surgical contexts, contributing to reduced postoperative pain and opioid consumption [4,8]. The lack of a statistically significant difference in pain scores in our study might be attributed to the overall effectiveness of both preoperative and postoperative ESPB, as well as the multimodal analgesic regimen employed.

Our findings, together with emerging evidence, indicate that preoperative ESPB may represent a valuable component of enhanced recovery after surgery (ERAS) protocols in minimally invasive biliary surgery. Its favorable safety profile, ease of administration, and intraoperative hemodynamic benefits facilitate perioperative workflows and improve patient outcomes [6]. Future large multicenter trials are warranted to confirm these advantages across diverse populations and to assess long-term impacts on recovery and healthcare resource utilization.

### Limitation

This study has some limitations. Being single-center and limited to ASA I–II patients undergoing elective laparoscopic cholecystectomy, its generalizability to higher-risk populations is restricted. Although patients were blinded, the anesthesiologist knew group allocation, which may have introduced bias. The absence of a no-block control group also precludes direct comparison with no regional intervention. Moreover, only short-term outcomes were assessed, and long-term effects remain unknown. Finally, the hemodynamic benefits of preoperative ESPB should be interpreted cautiously given potential confounders. Larger multicenter double-blind studies are needed to validate these findings.

To further contextualize our findings, recent advancements in regional anesthesia for LC include comparisons of ESPB with other truncal blocks like transversus abdominis plane (TAP) block and quadratus lumborum (QL) block. A recent meta-analysis by Zewdu, D. et al. [23] indicated that, while all three blocks provide effective analgesia, ESPB might offer superior visceral pain coverage due to its more medial spread. Another randomized controlled trial by Khalil, MS. et al. [24] compared preoperative ESPB with TAP block in LC and found similar analgesic efficacy but noted a more favorable hemodynamic profile with ESPB. These studies, along with ours, contribute to a growing body of evidence supporting the role of ESPB in multimodal analgesia for LC.

## 5. Conclusions

Preoperative and postoperative ESPBs offer comparable analgesic efficacy and opioid-sparing effects in LC. However, preoperative ESPB provides enhanced intraoperative hemodynamic stability and avoids the logistical challenges of performing blocks under anesthesia, including repositioning-related risks. These findings suggest that preoperative ESPB may be considered for integration into enhanced recovery after surgery (ERAS) protocols for minimally invasive biliary surgery, pending further large-scale multicenter trials.

## Figures and Tables

**Table 1:** Baseline demographic and clinical characteristics stratified by study group.

		Group 1 (*n*:40)	Group 2 (*n*:40)	*p*	*SMD*
Age (years)		45.4 ± 14.2	48.0 ± 13.6	0.413	0.18
BMI (kg/m^2^)		26.1 ± 3.92	27.6 ± 3.00	0.054	0.42
		***n*** **(%)**	***n*** **(%)**	***p***	
Sex	Female	29 (72.5%)	28 (70%)	0.805	0.06
	Male	11 (27.5%)	12 (30%)		
Comorbidity	Present	23 (57.5%)	31 (77.5%)	0.171	0.41
	Absent	17 (42.5%)	9 (22.5%)		
	Allergic Rhinitis	1 (2.5%)	0 (0.0%)	1.000	0.07
	Asthma + Diabetes Mellitus	0 (0.0%)	2 (5.0%)	0.494	0.22
	Asthma + Hypertension	0 (0.0%)	1 (2.5%)	1.000	0.16
	Diabetes Mellitus	1 (2.5%)	4 (10.0%)	0.363	0.29
	Diabetes Mellitus + Hypertension	4 (10.0%)	2 (5.0%)	0.673	0.18
	Hypertension	4 (10.0%)	5 (12.5%)	1.000	0.09
	Asthma	1 (2.5%)	3 (7.5%)	0.615	0.22
	Coronary Artery Disease	1 (2.5%)	1 (2.5%)	1.000	0.00
	Facial Paralysis	1 (2.5%)	1 (%)	1.000	0.00

Data are presented as mean ± standard deviation for continuous variables and as frequency (%) for categorical variables. Group comparisons were performed using independent *t*-tests or chi-square tests as appropriate. No significant differences were detected between groups across baseline variables.

**Table 2:** Temporal comparison of postoperative NRS pain scores by ESPB timing.

Postoperative Time	Group 1 (Mean ± SD)	Group 2 (Mean ± SD)	*p* Value
**0 min**	3.30 ± 2.63	4.25 ± 2.54	0.105
**2 h**	3.33 ± 2.08	3.40 ± 1.91	0.867
**4 h**	2.75 ± 1.43	3.13 ± 1.49	0.254
**6 h**	3.25 ± 1.69	3.38 ± 1.94	0.760
**12 h**	3.02 ± 1.56	3.58 ± 1.91	0.162
**24 h**	3.14 ± 0.07	3.48 ± 1.91	0.658

Values are presented as mean ± standard deviation. NRS scores were assessed at six time points postoperatively. No statistically significant differences were observed between groups at any time point (*p* > 0.05).

**Table 3:** Effect of ESPB timing on intraoperative hemodynamic parameters (mean ± SD).

Time Point		Group 1 (*n* = 40) (Mean ± SD)	Group 2 (*n* = 40) (Mean ± SD)	*p*
Baseline (0 min)	HR	81.3 ± 12.9	84.0 ± 15.4	0.395
	SBP	109.0 ± 21.5	114.0 ± 21.6	0.266
	DBP	69.1 ± 14.4	71.0 ± 16.2	0.586
	MAP	82.4 ± 16.2	84.9 ± 16.7	0.488
15 min	HR	80.4 ± 12.6	82.7 ± 15.5	0.468
	SBP	107.0 ± 24.4	114.0 ± 28.6	0.284
	DBP	70.3 ± 16.0	73.0 ± 18.4	0.473
	MAP	82.5 ± 18.3	86.5 ± 21.3	0.372
30 min	HR	77.9 ± 10.4	78.9 ± 12.0	0.692
	SBP	107.0 ± 19.0	117.0 ± 18.8	**0.016** *
	DBP	67.9 ± 12.1	73.5 ± 11.4	**0.036** *
	MAP	80.8 ± 13.8	88.0 ± 12.7	**0.018** *
60 min	HR	77.3 ± 11.7	77.1 ± 10.5	0.956
	SBP	102.0 ± 13.7	119.0 ± 19.6	**0.009** *
	DBP	66.6 ± 9.39	71.5 ± 10.0	0.153
	MAP	78.0 ± 10.3	87.3 ± 12.0	**0.023** *

Intraoperative systolic, diastolic, and mean arterial pressure (MAP) and heart rate values in Group 1 (preoperative ESPB) and Group 2 (postoperative ESPB). Group 1 exhibited significantly more stable MAP values at 30 and 60 min after incision and at extubation (*p* < 0.05), while no significant differences were observed in heart rate. (* *p* < 0.05, statistically significant).

**Table 4:** Effect of ESPB timing on postoperative hemodynamic parameters (mean ± SD).

Time Point		Group 1 (*n* = 40) (Mean ± SD)	Group 2 (*n* = 40) (Mean ± SD)	*p*
0 min	HR	87.7 ± 12.1	84.6 ± 12.3	0.258
	SBP	122.0 ± 15.7	134.0 ± 23.3	**0.005** *
	DBP	76.1 ± 12.2	81.5 ± 12.4	0.056
	MAP	91.2 ± 12.1	99.1 ± 14.5	**0.011** *
2 h	HR	82.8 ± 11.0	80.5 ± 11.5	0.376
	SBP	118.0 ± 13.5	121.0 ± 17.6	0.391
	DBP	75.0 ± 9.56	72.7 ± 10.1	0.300
	MAP	89.3 ± 10.2	88.6 ± 11.7	0.788
4 h	HR	81.3 ± 11.2	80.6 ± 8.30	0.735
	SBP	115.0 ± 13.9	113.0 ± 14.0	0.678
	DBP	71.1 ± 8.21	71.4 ± 8.33	0.882
	MAP	85.4 ± 9.75	85.5 ± 9.71	0.976
6 h	HR	82.4 ± 11.6	80.8 ± 7.49	0.472
	SBP	113.0 ± 14.4	114.0 ± 12.8	0.629
	DBP	70.7 ± 8.74	70.7 ± 8.73	0.980
	MAP	84.7 ± 9.90	85.0 ± 9.09	0.891
12 h	HR	81.1 ± 10.7	78.8 ± 13.8	0.413
	SBP	112.0 ± 14.9	115.0 ± 11.7	0.305
	DBP	69.9 ± 9.23	70.5 ± 8.01	0.767
	MAP	83.8 ± 10.5	85.2 ± 8.20	0.502
24 h	HR	80.9 ± 10.2	81.0 ± 8.54	0.953
	SBP	110.0 ± 13.1	113.0 ± 14.3	0.441
	DBP	68.0 ± 8.64	69.0 ± 9.11	0.616
	MAP	82.0 ± 9.35	83.7 ± 10.2	0.438

Postoperative systolic, diastolic, and mean arterial pressure (MAP) and heart rate values at 0 min (PACU), 2, 4, 6, 12, and 24 h. No significant differences were found between Group 1 (preoperative ESPB) and Group 2 (postoperative ESPB) at any time point (*p* > 0.05 for all comparisons). (* *p* < 0.05, statistically significant).

**Table 5:** Influence of ESPB administration timing on rescue analgesic requirements and tramadol consumption in the first 24 h.

Postoperative Time	Supplemental Analgesia	Group 1		Group 2		*p* Value
		*n*	%	*n*	%	
0 min	Present	9	22.5%	13	32.5%	0.317
	Absent	31	77.5%	27	67.5%	
2 h	Present	12	30%	15	37.5%	0.478
	Absent	28	70%	25	62.5%	
4 h	Present	7	17.5%	9	22.5%	0.576
	Absent	33	77.5%	31	82.5%	
6 h	Present	11	27.5%	12	30%	0.805
	Absent	29	62.5%	28	70%	
12 h	Present	11	27.5%	13	32.5%	0.626
	Absent	29	72.5%	27	67.5%	
24 h	Present	7	17.5%	8	20%	0.775
	Absent	33	82.5%	32	80%	
**24 h Tramadol Requirement**						
Tramadol (mg)		30.0 ± 45.9		22.5 ± 41.8		0.062

Values are presented as frequency (%) for categorical outcomes and mean ± standard deviation for continuous data. No statistically significant differences were observed between groups in the need for supplemental analgesia or tramadol consumption across all evaluated time points. Statistical comparisons were made using the chi-square test for categorical variables and the independent *t*-test for continuous variables.

**Table 6:** Likert-based assessment of patient satisfaction 24 hours after ESPB.

Likert Scale (1–5)		Group 1		Group 2	
**Patient Satisfaction Score** **(1 = Very Dissatisfied;** **5 = Very Satisfied)**		***n***	**%**	***n***	**%**
	**Very Dissatisfied (1)**	0	0.0%	1	2.5%
	**Dissatisfied (2)**	0	0.0%	3	7.5%
	**Neutral (3)**	10	25.0%	10	25.0%
	**Satisfied (4)**	25	62.5%	20	50.0%
	**Very Satisfied (5)**	5	12.5%	6	15%
	*p*-value	0.191			

Patient satisfaction was evaluated using a 5-point Likert scale at 24 h postoperatively. Values are presented as number and percentage (%). Statistical comparison was performed using the chi-square test. No significant differences were observed between groups (*p* > 0.05).

## Data Availability

The datasets used and/or analyzed during the current study are available from the corresponding author on reasonable request.

## Abbreviations

ESPB	Erector Spinae Plane Block
LC	Laparoscopic Cholecystectomy
ERAS	Enhanced Recovery After Surgery
NRS	Numeric Rating Scale
PACU	Post-Anesthesia Care Unit
MAP	Mean Arterial Pressure
HR	Heart Rate
SBP	Systolic Blood Pressure
DBP	Diastolic Blood Pressure
ASA	American Society of Anesthesiologists
EtCO_2_	End-Tidal Carbon Dioxide
SD	Standard Deviation
LAST	Local Anesthetic Systemic Toxicity
